# Oxidation State and Structure of Fe in Nontronite: From Oxidizing to Reducing Conditions

**DOI:** 10.1021/acsearthspacechem.3c00136

**Published:** 2023-09-27

**Authors:** Yanting Qian, Andreas C. Scheinost, Sylvain Grangeon, Jean-Marc Greneche, Alwina Hoving, Eric Bourhis, Nicolas Maubec, Sergey V. Churakov, Maria Marques Fernandes

**Affiliations:** † Laboratory for Waste Management, Paul Scherrer Institut, CH-5232 Villigen, Switzerland; ‡ Institute for Geological Sciences, University of Bern, CH-3012 Bern, Switzerland; § The Rossendorf Beamline at the European Synchrotron Radiation Facility (ESRF), Avenue des Martyrs 71, 38043, Grenoble, France; ⊥ Helmholtz Zentrum Dresden Rossendorf, Institute of Resource Ecology, Bautzner Landstrasse 400, 01328, Dresden, Germany; ¶ BRGM − French Geological Survey, 45060 Orléans, France; □ Institut des Molécules et Matériaux du Mans IMMM UMR CNRS 6283, Le Mans Université, 72085, Le Mans Cedex 9, France; ■ TNO Geological Survey of The Netherlands, P.O. Box 80015, 3508 TA Utrecht, The Netherlands; ▼ Interfaces, Confinement, Matériaux et Nanostructures (ICMN), CNRS/Université d’Orléans, UMR 7374, 1b rue de la Férollerie, CS 40059, 45071 Orléans, France

**Keywords:** Nontronite, Iron reduction, Structure, Fe redox state, Quantitative spectroscopy measurement

## Abstract

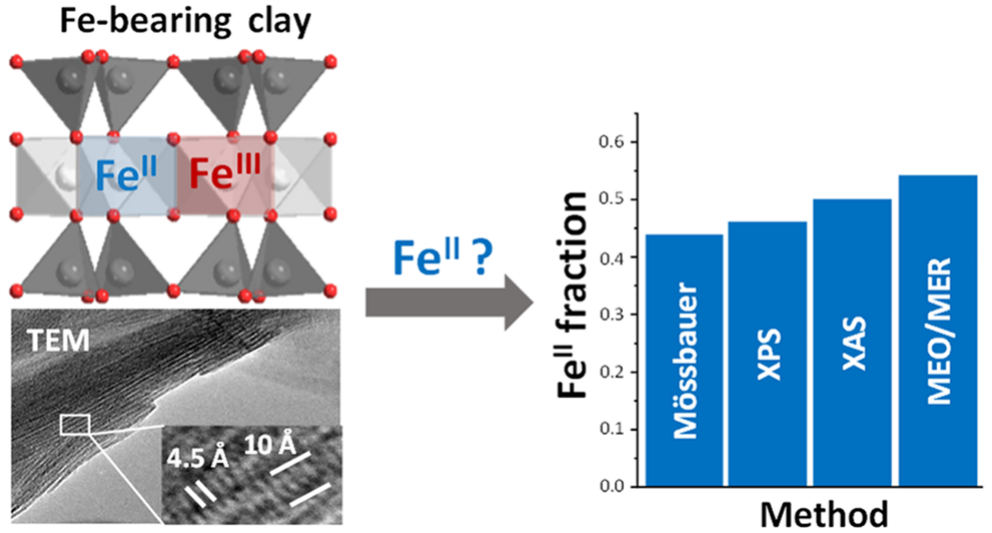

The redox reaction between natural Fe-containing clay minerals and its sorbates is a fundamental process controlling the cycles of many elements such as carbon, nutrients, redox-sensitive metals, and metalloids (e.g., Co, Mn, As, Se), and inorganic as well as organic pollutants in Earth’s critical zone. While the structure of natural clay minerals under oxic conditions is well-known, less is known about their behavior under anoxic and reducing conditions, thereby impeding a full understanding of the mechanisms of clay-driven reduction and oxidation (redox) reactions especially under reducing conditions. Here we investigate the structure of a ferruginous natural clay smectite, nontronite, under different redox conditions, and compare several methods for the determination of iron redox states. Iron in nontronite was gradually reduced chemically with the citrate-bicarbonate-dithionite (CBD) method. ^57^Fe Mössbauer spectrometry, X-ray photoelectron spectroscopy (XPS), X-ray absorption near edge structure (XANES) spectroscopy including its pre-edge, extended X-ray absorption fine structure (EXAFS) spectroscopy, and mediated electrochemical oxidation and reduction (MEO/MER) provided consistent Fe(II)/Fe(III) ratios. By combining X-ray diffraction (XRD) and transmission electron microscopy (TEM), we show that the long-range structure of nontronite at the highest obtained reduction degree of 44% Fe(II) is not different from that of fully oxidized nontronite except for a slight basal plane dissolution on the external surfaces. The short-range order probed by EXAFS spectroscopy suggests, however, an increasing structural disorder and Fe clustering with increasing reduction of structural Fe.

## 1 Introduction

In the critical zone, Fe(hydr)oxide minerals and Fe-bearing phyllosilicates play a fundamental role in controlling the mobility and bioavailability of several elements and molecules.^1^ While alternating redox conditions induced e.g. by fluctuations in the water table or by cycles of organic matter input/degradation often cause the (complete) dissolution and reprecipitation of Fe(hydr)oxides, structural Fe in phyllosilicate may change its oxidation state repeatedly while maintaining essentially the structural integrity of its host phase.^2^ Thereby, Fe-bearing clay minerals act as redox batteries, playing important roles e.g. in the natural carbon cycle, for nutrient availability, and for the mobility and toxicity of organic and inorganic pollutants.^3,4^ Ferric iron in minerals potentially limits organic matter mineralization in floodplains and thus sustains the ecosystem.^5,6^ Fe-bearing clay minerals efficiently retain pesticides,^7,8^ phosphates,^9,10^ organic contaminants,^11−13^ and heavy metals.^14−17^ Fe-bearing clay minerals used as engineered barriers and backfill in radioactive waste repositories are critical for immobilizing redox sensitive radionuclides, such as uranium,^18−22^ neptunium,^23,24^ technetium,^25−28^ and selenium.^29,30^ Iron redox reactions are involved in all these processes. The Fe(II)/Fe(III) ratio in clay minerals, its structural position, and the ability to participate in redox-reactions are the key parameters necessary for the understanding and modeling the redox-controlled retention processes.^31^ In contrast to iron (hydr)oxides, the geochemical behavior of natural Fe-bearing clay minerals is much less understood due to their diversity in composition and structure. In this study, we combine several microscopic and spectroscopic techniques to shed light on the behavior of iron in a typical Fe-rich smectite clay mineral, nontronite, under varying redox conditions.

Nontronite has a 2:1 layer structure, and each layer is composed of an octahedral (O) sheet sandwiched between two tetrahedral (T) sheets, known as the TOT layer. In dioctahedral clays, two-thirds of the octahedral sites are occupied by mostly trivalent cations, while in trioctahedral clays, all octahedral sites are occupied by mostly divalent cations. Octahedra with hydroxyl groups on opposing sides are labeled trans (M1) sites, while octahedra with hydroxyl groups on the same sides are labeled cis (M2) sites. Nontronite belongs to the dioctahedral group of Fe-bearing clay minerals;^32^ hence one-third of the cation sites in the octahedral sheets are vacant. The distribution of octahedral cations and vacancies in these sheets generates different octahedral orderings and thus different physical and chemical properties of the clay minerals.^33^ Therefore, a well-defined clay structure is the basis for any clay relevant research. While the octahedral ordering has been studied in the past, it is still debated because of the difficulty to obtain accurate structural configurations by experimental methods like TEM and XRD due to nanoscale particle size and the turbostratic stacking of the layers of these swelling clays. Based on ^57^Fe Mössbauer, infrared and X-ray absorption spectroscopies, as well as X-ray diffraction,^34−41^ and theoretical simulation,^42^ it has been established that octahedral sheets of Fe-rich clays are trans-vacant (M1 vacant or centrosymmetric structure), while the octahedral sheets of Fe-poor clays are cis-vacant (M2 vacant or noncentrosymmetric structure).^33^

In addition to octahedral ordering, Fe reduction in the clay requires charge compensation mechanisms to balance the decreased positive charge, which may lead to additional structural changes. Stucki et al. proposed a structural dehydroxylation induced by the pH-buffered dithionite reduction method^43^ and observed Fe, Si, and Al dissolution during clay reduction.^44^ Manceau et al. stated that Fe remains in 6-fold coordination as it is reduced to Fe(II) and that the Fe(II) cations migrate from cis-sites to neighboring empty trans-sites and possibly form trioctahedral clusters in the otherwise dioctahedral nontronite structure.^45^ The mechanisms have been linked to local structure instabilities followed by structural rearrangements. Such structural change is likely to alter octahedral ordering, but no direct evidence of the structural transformation has yet been reported, and little is known about the influence of the degree of clay reduction on clay surfaces and structures.

Powerful structural characterization methods are a prerequisite to investigate such structural alterations. Powder X-ray diffraction (XRD) provides structural, textural, and morphological information on mineral phases. Due to the presence of turbostratism, XRD patterns of swelling clays show only 00*l* and unresolved *hk* reflections. While this can be quantitatively accounted for in powder XRD, this is less easy in single crystal XRD. In contrast to the long-range order sensitivity of XRD, Extended X-ray absorption fine structure (EXAFS) spectroscopy probes the short-range order around the absorbing element within a few Angstrom distance. Extended transmission electron microscopy (TEM) is used to visualize the morphology and microstructure in clay minerals. Due to advances in resolution and sample preparation methods,^46^ it is now possible to acquire direct information on the structure of clay minerals at an atomic level.^47^ In addition to structural information and morphology, there is also a great demand for accurate and quantitative measurements of Fe(II) and Fe(III) content, especially for the studies of redox reactions. Currently, Mössbauer spectrometry, X-ray photoelectron spectroscopy (XPS), X-ray absorption near edge structure (XANES) spectroscopy, and mediated electrochemical oxidation and reduction (MEO/MER) have been developed for this purpose. Mössbauer spectroscopy probes the core of Fe, while XAS probes excitations from core to valence bands. The analysis by XPS is surface sensitive since restricted to the topmost 2–10 nm of a sample, while the hard X-rays employed for XANES and EXAFS penetrate even at the relatively low energy of the Fe K-edge (7.112 keV) tens of microns thereby probing typically the bulk of a sample; MEO/MER measures the electron donating and accepting capacities of the sample through chemical reactions. Each method has its strengths and limitations, but a direct benchmarking of the different methods for Fe(II)/Fe(III) quantification has to the best of our knowledge never been performed.

In this study, we conducted clay reduction experiments in the aqueous phase and applied XRD, TEM, Mössbauer spectrometry, XPS, XANES, EXAFS and MEO/MER to (1) study the clay dissolution in the reduction process, (2) reveal the structure of nontronite, and (3) compare the results of four spectroscopic and one chemical method for the measurement of the Fe redox state as a function of increasing reduction by CBD. Based on this analysis, we provide a reliable structural characterization of nontronite and provide reference guidelines for the redox state measurements of iron in clay minerals.

## 2 Materials and Methods

All clay preparation and reduction steps as well as the preparation of samples for the different analytical methods were conducted in an anoxic glovebox under controlled N_2_ atmosphere (O_2_ < 0.1 ppm).

### 2.1 Clays and Clay Reduction by Citrate-Bicarbonate-Dithionite

The nontronite clay NAu-2 was obtained from the Clay Source Repository of the Clay Minerals Society (Purdue University, West Lafayette, IN). The chemical composition in wt % of NAu-2 raw material given by the Clay Minerals Society is SiO_2_ 56.99, Al_2_O_3_ 3.4, Fe_2_O_3_ 37.42, MgO 0.34, CaO 2.67, Na_2_O 0.11, K_2_O 0.02, and its chemical formula is (M^+^_0.97_)[Si_7.57_Al_0.01_Fe_0.42_][Al_0.52_ Fe_3.32_ Mg_0.7_]O_20_(OH)_4._^48^ According to several sources, the Fe content varies between 21 and 38 wt %.^49^ In our study, the Fe content of native NAu-2 is 22 wt % as determined by X-Ray Fluorescence (XRF). The raw clay was subjected to washing, purification, and sedimentation treatments to obtain a homogeneous, single-phased Na-form clay suspension with ≤ 0.5 μm Stokes diameter. The treatment includes the following steps: peptization with deionized water, cation exchange with 1 M NaCl to obtain the Na form, pre-equilibriation in the respective clay-water, centrifugation to obtain particles with a Stokes radius ≤ 0.5 μm, flocculation with 1 M NaCl, removal of acid soluble impurities by an acid treatment at pH 3, and finally conversion to 0.1 M NaCl background electrolyte. This treatment largely follows the method published by Baeyens et al. except for replacing NaClO_4_ by NaCl.^50^

#### 2.1.1 Clay Dissolution Experiment

The purified nontronite was reduced by the citrate-bicarbonate-dithionite (CBD) method essentially following Stucki et al., however without heating.^51^ Different degrees of reduction were obtained by adding different amounts of dithionite, while all other experimental conditions were kept the same. 7 mL of nontronite clay suspension (dry weight: 20.29 g/L) was first mixed with a citrate-bicarbonate (CB) buffer solution (0.4 mL of 0.5 M Na_3_C_6_H_5_O_7_·2H_2_O and 5.6 mL of 1 M NaHCO_3_) in centrifuge tubes for about an hour. A total of fourteen samples with varying degrees of reduction were obtained by adding the following amounts of dithionite (Na_2_S_2_O_4_): 0 g, 0.01 g, 0.02 g, 0.05 g, 0.1 g, 0.15 g, 0.2 g, 0.25 g, 0.3 g, 0.35 g, 0.43 g, 0.55 g, 0.8 g, 1.0 g. The tubes were filled with degassed 0.1 M NaCl solution up to 24 mL. The reduction process took about 24 h under continuous upside-down shaking. The clay suspensions were then centrifuged without an additional washing step to separate the clay fraction from the supernatant, which was collected for ICP-OES measurement at neutral pH. The absolute amount of element X dissolved (*X*_dis_) (μmol/g) was calculated according to eq 1. The percentage of normalized element X released (*X*_%_) (%) was calculated with eq 2.
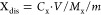
1
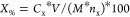
2where *C*_x_ (ppm or mg/L) is the concentration of dissolved element X in the supernatant measured by ICP-OES; *V* (L) is the volume of the solution; *M* (kg/mol) is the molar mass of X; *m* (g) is the mass of the clay mineral; *n*_x_ is the total mole amount (μmol) of X in nontronite.

#### 2.1.2 Low-Reduced and High-Reduced NAu-2 Samples

Larger quantities were prepared at only two degrees of reduction for the different characterization methods, following the same reduction method as before.^51^ Two airtight glass bottles with 500 mL nontronite clay suspension (dry weight: 18.09 g/L, 9 g clay in total, 0.03 mol Fe) were first mixed with a CB-buffer solution (12 mL of 0.5 M Na_3_C_6_H_5_O_7_·2H_2_O and 163 mL of 1 M NaHCO_3_) for about an hour. After equilibrium with CB-buffer, the dithionite powder was added to the clay suspension. For the “high-reduced” sample (high-red NAu-2), we added 27 g of dithionite, which represents three times the mass of clay, corresponding to a molar ratio S/Fe of 10. For the “low-reduced” sample (low-red NAu-2), we added 1.3 g of dithionite, which corresponds to a molar ratio S/Fe = 0.5. The bottles were then filled with degassed 0.1 M NaCl solution to a volume of 700 mL. The clay suspensions were stirred for one day. After the reduction step, the clay suspensions were transferred into dialysis bags and washed first with 1 M NaCl at pH 4 to remove the added chemicals and dissolved elements which could be readsorbed onto the clay surface, and then with 0.1 M NaCl without pH adjustment to bring the clay suspension to a 0.1 M NaCl matrix. The washing process was considered to be complete when the concentrations of citrate and bicarbonate in the conditioning solution were lower than 10^–9^ mol/L. Reduced clay suspensions were stored in airtight glass bottles covered by aluminum foil in the glovebox. High-red NAu-2 was blue-green and low-red NAu-2 was green after reduction (Figure S1). Even though the glovebox atmosphere contained still up to 0.1 ppm O_2_, the limited headspace of the bottles would restrict reoxidation of structural Fe in the clay to 2.4 × 10^–7^%.

### 2.2 X-ray Diffraction

All the sample preparation steps were conducted in the glovebox. The clay suspensions were filtered (cut-off diameter of 0.1 μm), washed with ethanol, and crushed with a mortar to produce a powder of dry material that was inserted in a polyimide capillary (internal diameter: 1.47 mm; wall thickness: 0.05 mm) and the capillary was sealed on both sides using wax. The airtight capillary was then taken out of the glovebox and loaded into a Bruker D8 Advance diffractometer equipped with a Mo anode (λ = 0.7107 Å) and a LynxEye SE detector. Diffraction data were collected in the 4–130° 2θ range, in continuous scan mode, and averaged every 0.03° 2θ. Total collection time was 23 h. No color change was observed between the initial and final sample, confirming the conservation of the original Fe oxidation state during the measurement. To further check that the sample preparation did not lead to partial sample oxidation, the same procedure was applied to a green rust samples with very fast oxidation kinetics, even at low *P*_O2_, and no change in color and XRD pattern was observed between the initial sample and the same after this sample collection procedure (data not shown).

### 2.3 Transmission Electron Microscopy

Transmission electron microscopy (TEM) experiments were performed using a Philips CM 20 operated at 200 kV. The clay suspensions were filtered in the glovebox (cut-off diameter of 0.1 μm), washed with ethanol, and crushed with a mortar to produce a powder of dry material, which was embedded in resin (Agar 100) and left for polymerization at 60 °C for 1–2 days, under anoxic conditions. The hardened resin was then sliced to produce 60–80 nm thick sections that were mounted on lacey carbon films loaded on Cu grids. This procedure was previously applied to study samples prone to Fe oxidation (feitknechite,^52^ green rust^53^). In these studies, no trace of oxidation was observed.

### 2.4 ^57^Fe Mössbauer Spectrometry

Mössbauer spectra were recorded at 300 and 77 K, using a constant acceleration spectrometer (driving unit supplied by WissEl GmbH, Germany), a ^57^Co source dispersed in a Rh matrix and a bath cryostat. The velocity of the spectrometer was calibrated using an α-Fe foil at 300 K. The Mössbauer sample, consisting of a thin layer containing 5 mg/cm^2^ Fe, was prepared in the glovebox and the holder then sealed for transport to the instrumental facilities. The sample holder was under He gas at 77 K and in vacuum at 300 K during the measurement. Identical spectra were obtained during repeated measurements, hence sample oxidation during the measurements can be excluded. The values of the hyperfine parameters were refined using a least-squares fitting procedure (MOSFIT, unpublished software, Le Mans Université, France) with independent quadrupole doublets composed of Lorentzian lines. Isomer shift (I.S.) are reported relative to that of an α-Fe spectrum obtained at RT. Since the f-Lamb-Mössbauer factors, which correspond to the fraction of γ rays emitted and absorbed without recoil, are assumed to be identical for the different phases present in the samples and for the different Fe species present in the same phase, the proportions of each Fe species are proportional to the relative spectral area.

### 2.5 X-ray Photoelectron Spectroscopy

XPS measurements were performed in an ESCALAB Xi+ X-ray photoelectron spectrometer (ThermoScientific) employing a monochromated Al Kα Xray source (hν = 1486.6 eV). High-resolution spectra were collected using an analysis area of 650 × 650 μm^2^ and a 20 eV pass energy. The C(1s) level (284.8 eV) was taken as the reference binding energy. The sample was prepared by suspending an aliquot of the nontronite sample in ∼1 mL bidistilled water inside the glovebox, agitated by hand, and then pipetting an aliquot of 0.5 mL of the suspension onto a carbon sample holder. The suspension was then dried at 35 °C in an oven in the glovebox. The dried sample was sealed in an airtight jar, itself sealed in an airtight aluminum bag. It was then taken out of the glovebox and opened just before being positioned in the measurement apparatus, where it was immediately subjected to vacuum conditions. A charge neutralizer was used for data collection, being monitored using the C(1s) signal corresponding to adventitious carbon.^54,55^ C(1s), Fe(2p), O(1s), Si(2p) spectra were collected and fitted using the Avantage software (ThermoScientific) via a Lorentzian–Gaussian peak with a default value of 30% Lorentzian contribution, and a smart background removal was used for all spectra.

### 2.6 XANES and EXAFS Sample Preparation and Spectra Collection

The Fe-K XANES main and pre-edge were recorded to determine directly the Fe oxidation state, while Fe-K EXAFS was measured to determine the short-range structure around Fe. For this, the clay suspension after reduction was centrifuged, the wet clay pastes were placed into a high density polyethylene (HDPE) double-confined sample holder in the glovebox (O_2_ < 0.1 ppm), and the holder was then heat-sealed. Once removed from the glovebox, the holders were immediately dropped into LN_2_ for flash freezing. The samples were stored and transported in a LN_2_-filled Dewar, and transferred in frozen state within less than 5 min into the He-cryostat at the beamline for measurements.

The measurements were carried out at the Rossendorf Beamline (ROBL) BM20 of the European Synchrotron Radiation Facility (ESRF), France.^56^ The storage ring was operated at 6 GeV in top-up mode with a ring current of 200 mA. The X-ray beam was monochromatized by a Si(111) double-crystal monochromator. Higher harmonics were suppressed by using two, 1.4 and 1.2 m long silicon mirrors with a grazing incidence angle of 2.5 mrad. Up to 8 samples were loaded at the same time in the closed-cycle He-cryostat (CryoVac) running at 10–15 K. The energy of the monochromator was calibrated with an Fe foil (7112 eV). The Fe K-edge XAS spectra were collected in fluorescence mode with an 18-discrete-elements Ge-detector (Mirion) equipped with a Falcon-X (XIA) electronic spectrometer using energy steps of 0.5 eV across the XANES region. The recorded raw data were first averaged in Sixpack^57^ and then processed in WinXAS^58^ using standard procedures for normalization, conversion into k-space by using the first inflection point of the main edge, and spline background removal with the autospline functionality of WinXAS. EXAFS spectra were extracted with k-weight 3 and k-range 2–12 Å. The EXAFS shell fitting was carried out in WinXAS in Fourier-transformed R space (k-range 2–12 Å^–1^; Bessel window; R-range 1–5.5 Å). Theoretical EXAFS paths for shell fitting were calculated self-consistently using FEFF9.6.4,^59^ based on the Garfield nontronite structural model.^60^

The pre-edge region of the Fe K-edge was analyzed with Origin software. A cubic spline interpolation was extended several eV before and after the pre-edge to subtract the background from the normalized XANES spectra.^61^ The such-derived, normalized pre-edge spectra were deconvoluted with two Voigt functions without any constraints. The centroid position is calculated according to eq 3.^62^
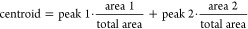
3

Principal component analysis (PCA) of XANES spectra was conducted with the Iterative Target Transformation Factor Analysis (ITFA) software package.^63^ Linear combination fits (LCF) were performed with Athena.^64^

### 2.7 Mediated Electrochemical Oxidation and Reduction

Electron donating and accepting capacities (EDC and EAC) were measured to determine the redox-active Fe in the NAu-2 samples. For this, an electrochemical setup was used as described in Gorski et al.^65^ The whole experiment was performed in the glovebox. The electrolyte in all experiments consisted of 0.1 M NaCl, buffered to pH 7 by 0.01 M MOPS (3-(*N*-morpholino)propanesulfonic acid). To facilitate the transfer of electrons between the clay particles and the working electrode, dissolved one-electron transfer mediators were added to the electrochemical cell. The maximum EDC was measured at an applied potential of 0.6 V vs standard hydrogen electrode (SHE) using ABTS (2,2′-azino-bis(3-ethylbenzothiazoline-6-sulfonic acid) as a mediator, and the maximum EAC was measured at an applied potential of −0.6 V vs SHE with TQ (1,1′-trimethylene-2,2′-bipyridyl) as a mediator. After equilibration of the mediator with the electrochemical cell at a specific applied potential, a small quantity of NAu-2 suspension (0.02 mL of ∼14 g/L) was added and the current monitored. The oxidative and reductive current peaks, caused by oxidation or reduction of redox-active Fe in the clay mineral, were integrated resulting in the EDC or EAC in moles of electrons per gram of sample. Since only the redox active Fe is assessed by MEO/MER, the total Fe was determined by total reflection X-ray fluorescence (TXRF) (S2 PICOFOX, Bruker). A drop of the clay suspension together with an added internal standard (Se and Ge) was evaporated on a glass carrier disk which was then inserted in and measured by the TXRF.

## 3 Results and Discussion

### 3.1 Reduction

**Figure 1:**
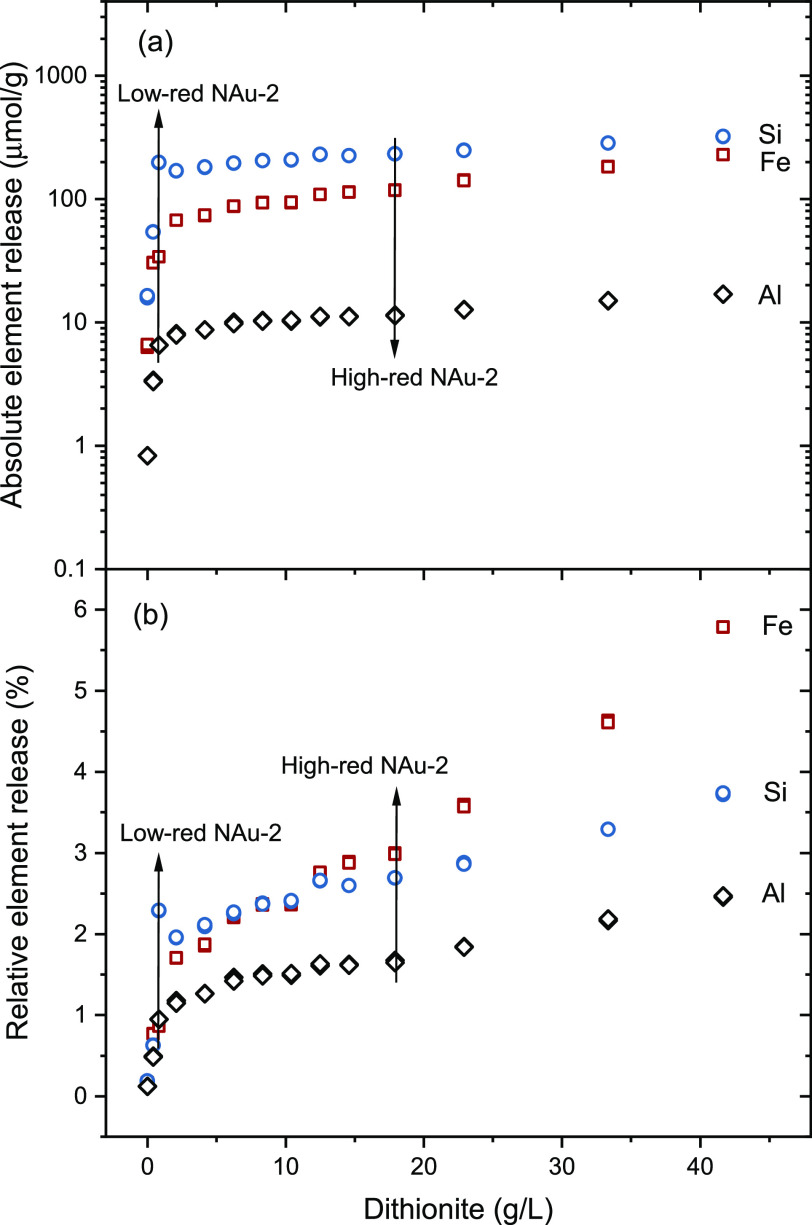
Si (blue circles), Fe (red squares), and Al (black diamonds) released from nontronite by CBD reduction as a function of increasing amounts of dithionite (errors are smaller than symbol size). Arrows mark the values for the two bulk samples low-red NAu-2 and high-red NAu-2 used for detailed analyses. (a) Absolute element release in μmol/g; (b) relative element release in percent.

Clay dissolution is widely observed during the CBD reduction process.^44,51,66^ We performed here a series of reduction experiments without a washing process in order to investigate the relationship between the degree of reduction and eventual dissolution of the clay by the reduction procedure. A blank experiment without adding dithionite is considered to represent the dissolution equilibrium of nontronite in 0.1 M NaCl matrix, with 0.8 μmol/g Al, 6.4 μmol/g Fe, and 16.1 μmol/g Si in the supernatant. The amount of Fe, Si, and Al released as a function of dithionite is illustrated in Figure 1. The dissolution of all three elements generally increases with dithionite indicating that the CBD treatment causes additional clay dissolution as already reported in previous studies.^51,66^ The released concentrations of Si, Al, and Fe are in the same order of magnitude as the ones reported by Jaisi et al.^66^ At the highest amount of dithionite added, 42 g/L, the released concentrations of Si, Fe, and Al are 322.1 μmol/g, 228.6 μmol/g, and 17.0 μmol/g, respectively, corresponding to 3.7%, 5.8%, and 2.5% dissolution with respect to their contents in NAu-2. Above 20 g/L dithionite, the release of Fe is increasing faster than that of Si and Al (Figure 1b), indicating a slight preferential dissolution of Fe up to 5.8% possibly due to a structural reorganization. This suggests that the reduced clay layer structure may not be representative anymore of the initial nontronite layer structure after harsher CBD treatments. This is in line with the work by Hadi et al.,^67^ where the negative layer charge of nontronite increased with the degree of reduction (Fe(III) to Fe(II)) at a lower reduction level but then dropped rapidly at a higher reduction level due to structural reorganization. Jaisi et al. observed similar findings, whereby the stability of the NAu-2 structure remained intact when the reduction degree was below 30%; however, the stability decreased without affecting the structural integrity as the reduction degree increased to 49% indicated by a consistent 001 peak position in XRD patterns, and ultimately, the NAu-2 structure transitioned into an amorphous state after reaching a Fe(III) reduction degree of 71%.^66^ Although no direct structural information can be derived from dissolution experiments, our results suggest that the structure of our two samples used for more detailed analyses, i.e., low-red NAu-2 (0.9% Fe dissolution, 19% Fe(III) reduction degree, Table 1) and high-red NAu-2 (3.0% Fe dissolution, 44% Fe(III) reduction degree, Table 1), remains identical to that of the initial nontronite NAu-2.^48^

### 3.2 X-ray Diffraction

**Figure 2:**
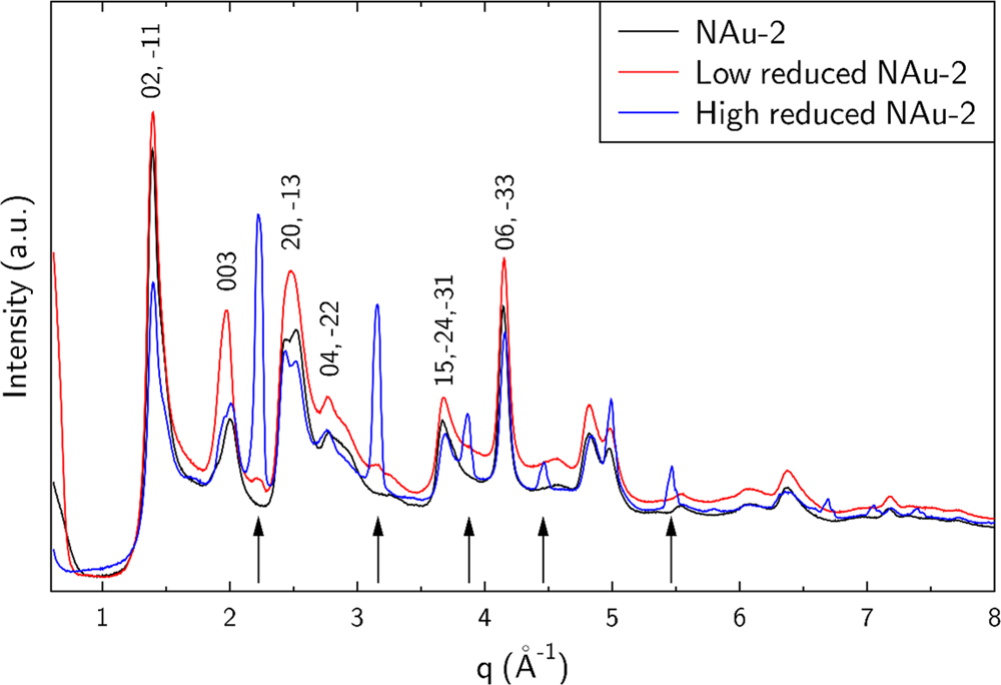
XRD patterns of native NAu-2, low-red NAu-2, and high-red NAu-2. Additional lines corresponding to KFeO_2_ are marked with arrows.

Samples of native NAu-2, washed low-red, and high-red NAu-2 were collected for XRD measurements (Figure 2). The XRD pattern of sample NAu-2 is characteristic for nontronite, with the main reflection being 00*l* reflections and asymmetrical *hk* reflections due to turbostratic stacking, for example, the (11, 02) and (20, 13) reflections at ∼1.41 Å^–1^ and ∼2.47 Å^–1^. With increasing reduction degree, additional reflections (black arrows) emerge, for example at 2.23 Å^–1^, 3.15 Å^–1^, and 3.86 Å^–1^, and are attributed to a phase structurally similar to KFeO_2_ (ICDD Card #83–-2153). The KFeO_2_-like phase was not observed in the as-prepared reduced NAu-2 suspension, strongly suggesting that it precipitates during the drying procedure, in spite of using a drying procedure optimized to avoid the possible precipitation of secondary phases. Besides this, the two reduced samples show the typical nontronite pattern, demonstrating the structural integrity after the two CBD treatments.

### 3.3 Transmission Electron Microscopy

**Figure 3:**
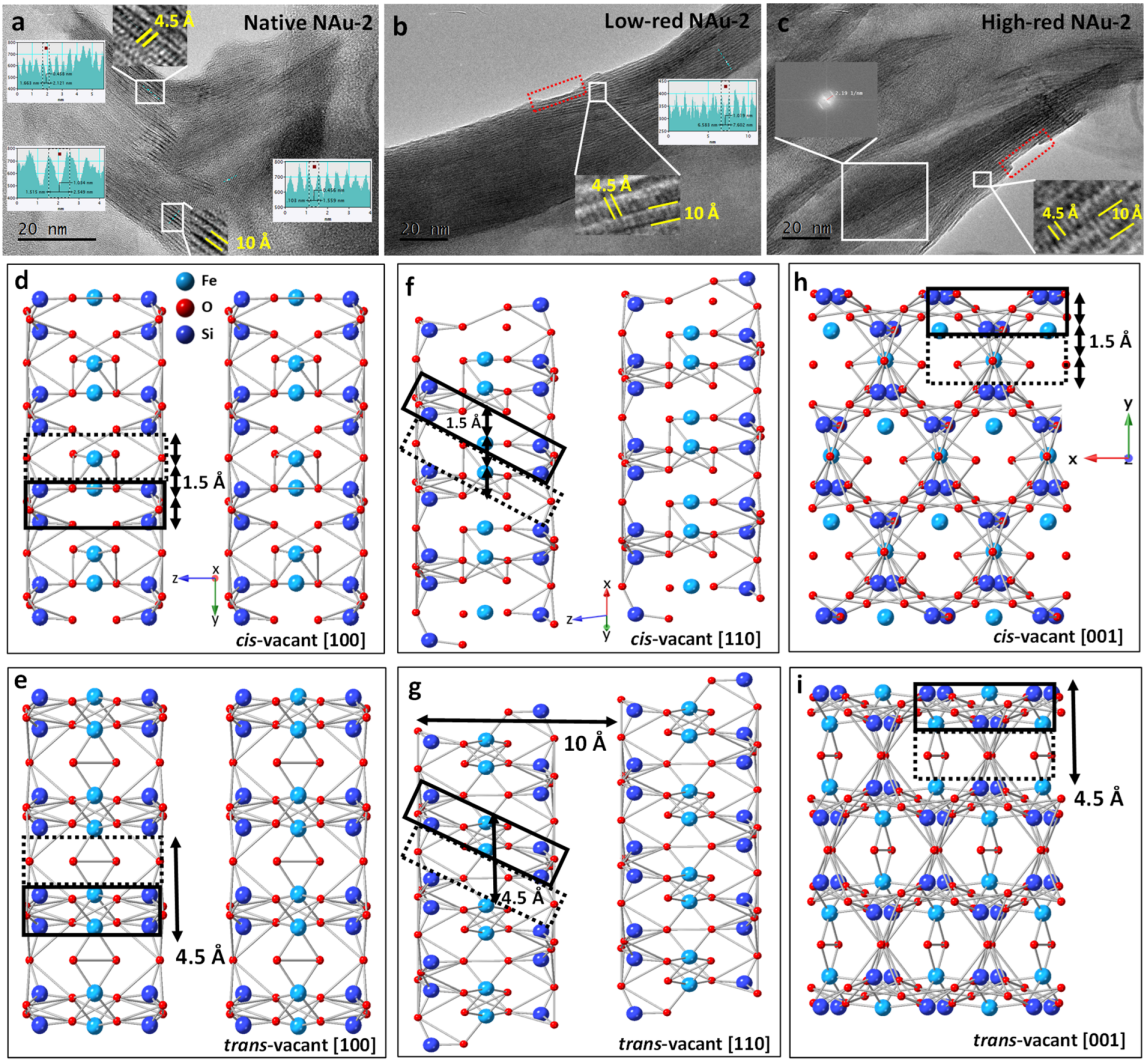
TEM image of (a) native NAu-2, (b) low-red NAu-2, (c) high-red NAu-2. Nontronite structure of (d) cis-vacant octahedra [100], (e) trans-vacant octahedra [100], (f) cis-vacant octahedra [110], (g) trans-vacant octahedra [110], (h) cis-vacant octahedra [001], (i) trans-vacant octahedra [001].

TEM allows studying the morphology and structure of clay minerals, including layer stacking, atomic arrangements, and surface site defects. With the specific sample preparation method employed here, the clay platelets where preferentially lying perpendicular to the electron beam (i.e., most particles had the *ab* plane perpendicular to the beam), which eased the imaging of the layer plane structure. Furthermore, this unified orientation allowed us to minimize exposure time, while longer exposition times would have been required if the sample had to be tilted to obtain an adequate observation angle. Finally, this preparation procedure allowed us to obtain numerous particles having the same orientation and about the same depth within the resin. Therefore, the acquisition parameters could be optimized first with a trial particle, before moving to a particle used for analysis. The layer structure of nontronite was observed in all three clay samples (Figure 3), and no other solids were detected (note that the two reduced samples were prepared with an additional acid wash after reduction to remove any impurities that have the potential to reabsorb to the clay mineral or remain as precipitates in the clay suspension). As highlighted by the yellow lines, the layer-to-layer distance is 10–12 Å, which agrees well with the theoretical layer-to-layer distance in smectite, derived by XRD of oriented clay films.^68^ Again, the reduced NAu-2 has the same layer structure and a similar layer-to-layer distance as the native sample, showing that the clay structure is stable and well preserved after the CBD treatment. Nevertheless, a slight dissolution of the basal planes is noticeable on the external surfaces but not in the bulk structure (see areas defined with red dotted lines in Figure 3b,c). The structure images obtained by TEM, therefore, corroborate the results of the reduction experiment (Figure 1) and XRD (Figure 2) in that the observed 2.8% dissolution affects only the external basal plane surfaces and not affect the bulk structure of nontronite.

In addition, another distance of 4.5 Å was observed in all three nontronite samples. In the TOT layer, a lattice fringe with a distance of 4.5 Å is clearly visible along each layer. The same value could also be obtained by applying the fast Fourier transform (FFT) method to the structure area of the selected layer in Figure 3c, to transfer the image in the selected region to the frequency domain. The reciprocal value of the frequency 2.19 1/nm is about 4.5 Å, and this distance exists throughout the whole image. This distance corresponds to trans-vacant octahedra in the [100] and [001] axis, where the lattice fringes of 4.5 Å and 10 Å are perpendicular (Figure 3a,c,e,i), or in the [110] axis with inclined lattice fringes (Figure 3b,g), where Fe and Si are located in the solid line region and only O occupies the area delineated by dashed lines. In contrast to the trans-vacant case, Fe and Si would be evenly distributed throughout the cis-vacant octahedra (Figure 3d,f,h); the resulting cation separation distance of ∼1.5 Å would be too small to be resolved at the available TEM settings. Only with a TEM resolution better than 1.5 Å, both cis-vacant and trans-vacant octahedra would be discernible.

In previous work, it was proposed that iron moves from *cis* to *trans* sites during reduction and forms trioctahedral domains and large vacancies in the octahedral sheets.^41 ,45 ,69 ,70^ We could not confirm this for the low-red NAu-2 and high-red NAu-2 samples. Either these phenomena are irregular on a small scale and hence difficult to capture by TEM, or our relatively mild CBD treatment did not cause such a reorganization of the octahedral sheets. Note that the degree of reduction of nontronite was more than 99% in the work of Manceau et al.,^45^ while it is less than 44% in our study. According to Figure 1, a reduction degree of more than 44% (>20 g/L dithionite) can cause preferential dissolution of Fe in the octahedral sheet, which may result in further iron migration and structural reorganization.^67^ Therefore, the relationship between structural reorganization and reduction degree beyond ∼40% reduction should be studied in more detail in future work.

### 3.4 Mössbauer Spectrometry

**Figure 4:**
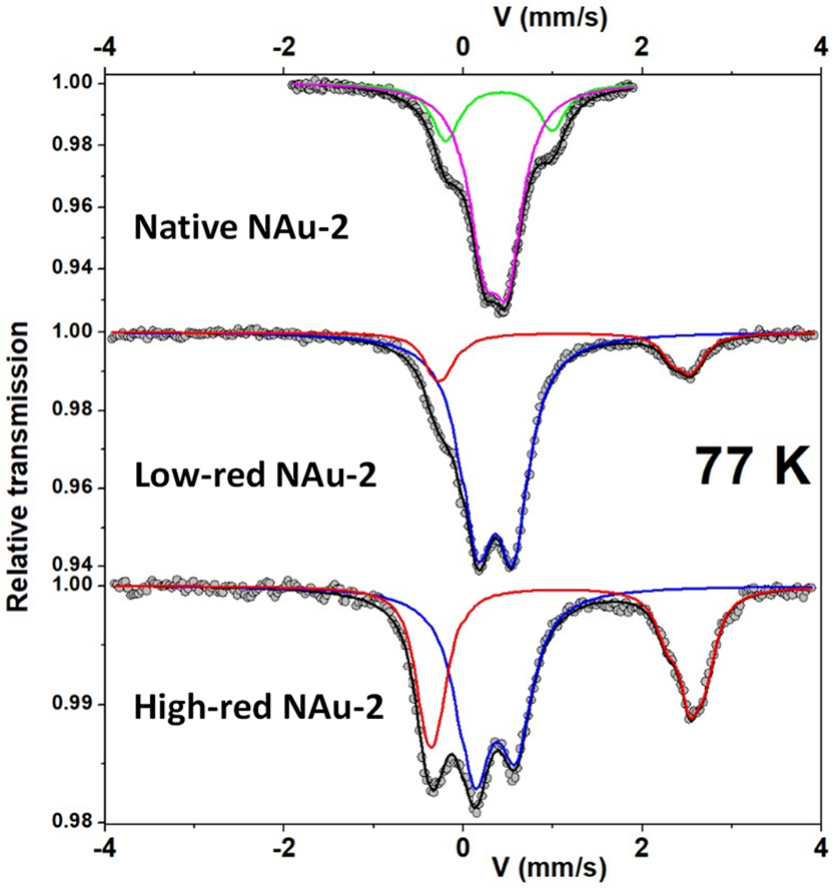
Mössbauer spectra of native NAu-2, low-red NAu-2, and high-red NAu-2 at 77 K (see Table 1 for the colors).

The measurements were first carried out with a large velocity scale to check for the presence of additional lines, i.e. magnetic sextets attributed to certain magnetic Fe oxides. Then, the measurements were repeated with a more appropriate velocity scale to optimize the resolution of the hyperfine structures in the region of interest. Only the Mössbauer spectra registered at 77 K (low velocity scale) are presented in Figure 4, and their refined values of the hyperfine parameters are listed in Table 1. The spectrum of the native NAu-2 required decomposition by at least two quadrupolar components, but a third component can be considered, further improving the fit. Another fitting model can be obtained by considering the presence of a certain degree of preferential orientation, in agreement with the previous results of X-ray diffraction in this study, with asymmetric quadrupolar components. These different models are characterized by two different values of the isomer shift which are attributed to high spin (HS) Fe(III) species located in cis- and trans-octahedral sites, as previously observed in the literature.^71^ It is also important to note that the presence of HS Fe(III) species in the tetrahedral site, if any, would not exceed 2% (limit of detection). The spectra obtained on low-red and high-red NAu-2 differ from the unreduced, native NAu-2 with the appearance of an additional broad line, whose intensity increases with the degree of reduction. Different fitting models were considered involving between two and six different quadrupolar components, associated or not with some preferential orientation, as previously mentioned in the XRD analysis. They lead to two subspectra, as shown in Figure 4, and the corresponding refined mean values of the hyperfine parameters are given in Table 1. They are unambiguously attributed to the presence of HS Fe(III) and HS Fe(II) species, the second component increasing with the degree of reduction. It is also important to note that the different fitting models do not allow us to confirm the presence of cis and trans HS Fe(III) located in octahedral units. According to these fits, the fraction of Fe(II) is 0 in native NAu-2, 0.19 in low-red NAu-2, and 0.44 in high-red NAu-2 (see a comparison of Fe(II) fractions obtained by all methods below).

**Table 1:** Refined Hyperfine Parameters for the Different Components of the 77 K Mössbauer Spectra^a^

	δ (mm/s) ± 0.01	QS (mm/s) ± 0.02	% ± 2	Fe site	color (see Figure 4)
Native NAu-2	0.52	1.17	24	Fe(III)	Green
	0.47	0.27	76	Fe(III)	Magenta
Low-red NAu-2	0.47	0.41	81	Fe(III)	Blue
	<1.23>	<2.73>	19	Fe(II)	Red
High-red NAu-2	0.47	0.45	56	Fe(III)	Blue
	<1.22>	<2.86>	44	Fe(II)	Red

a See Figure 4 for the color of spectral components.

### 3.5 X-ray Photoelectron Spectroscopy

**Figure 5:**
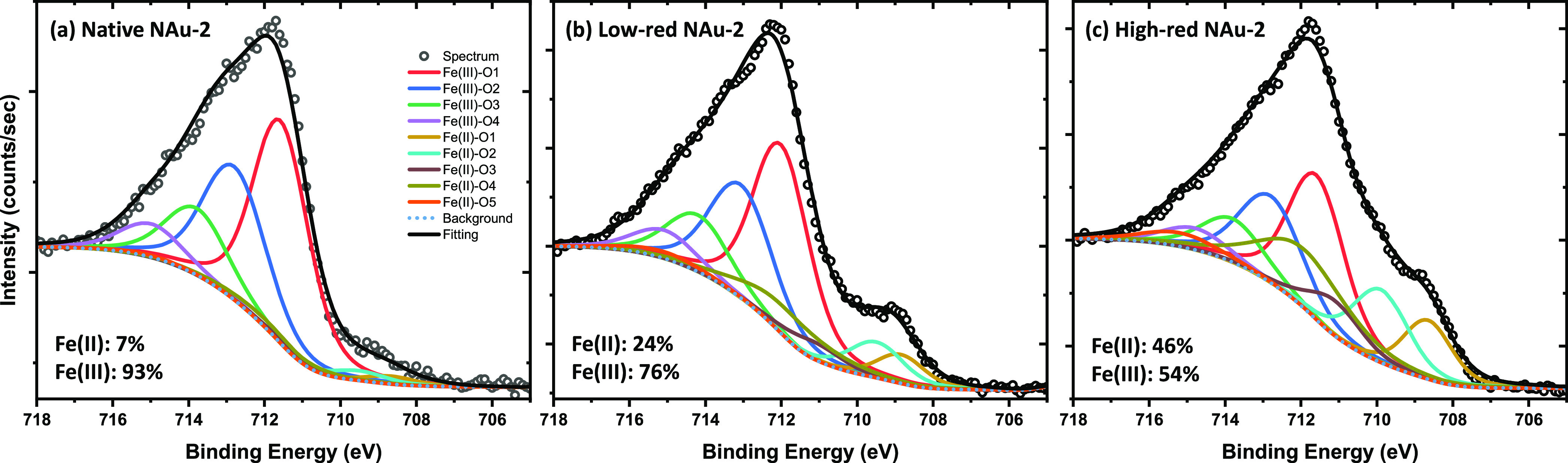
XPS spectra of (a) Native NAu-2; (b) Low-red NAu-2; (c) High-red NAu-2. The binding energy, full width at half-maximum (fwhm), area, and percentage of each Fe species are shown in Table S1.

XPS spectra were used to further probe the Fe redox state of nontronite samples. As shown in Figure 5, Fe(II) fractions in native NAu-2, low-red NAu-2, and high-red NAu-2 are 0.07, 0.24, 0.46, respectively, hence 5 to 10% higher than the values derived by Mössbauer spectrometry (Table 1). Such slightly higher Fe(II) fractions in nontronite derived by XPS have been explained by Yuan et al. with an accumulation of Fe(II) at the surface through electron transfer,^69^ which is selectively probed by the surface-sensitive XPS method.^72^ Since a reduction of U(VI) to U(IV) by X-ray irradiation in XPS measurement was observed,^73^ 7% of Fe(II) in native NAu-2 could also result from X-ray irradiation of Fe(III). The effect of X-ray irradiation is hard to avoid; therefore, caution has to be taken in interpreting XPS data quantitatively.

### 3.6 Fe K-Edge XAFS

**Figure 6:**
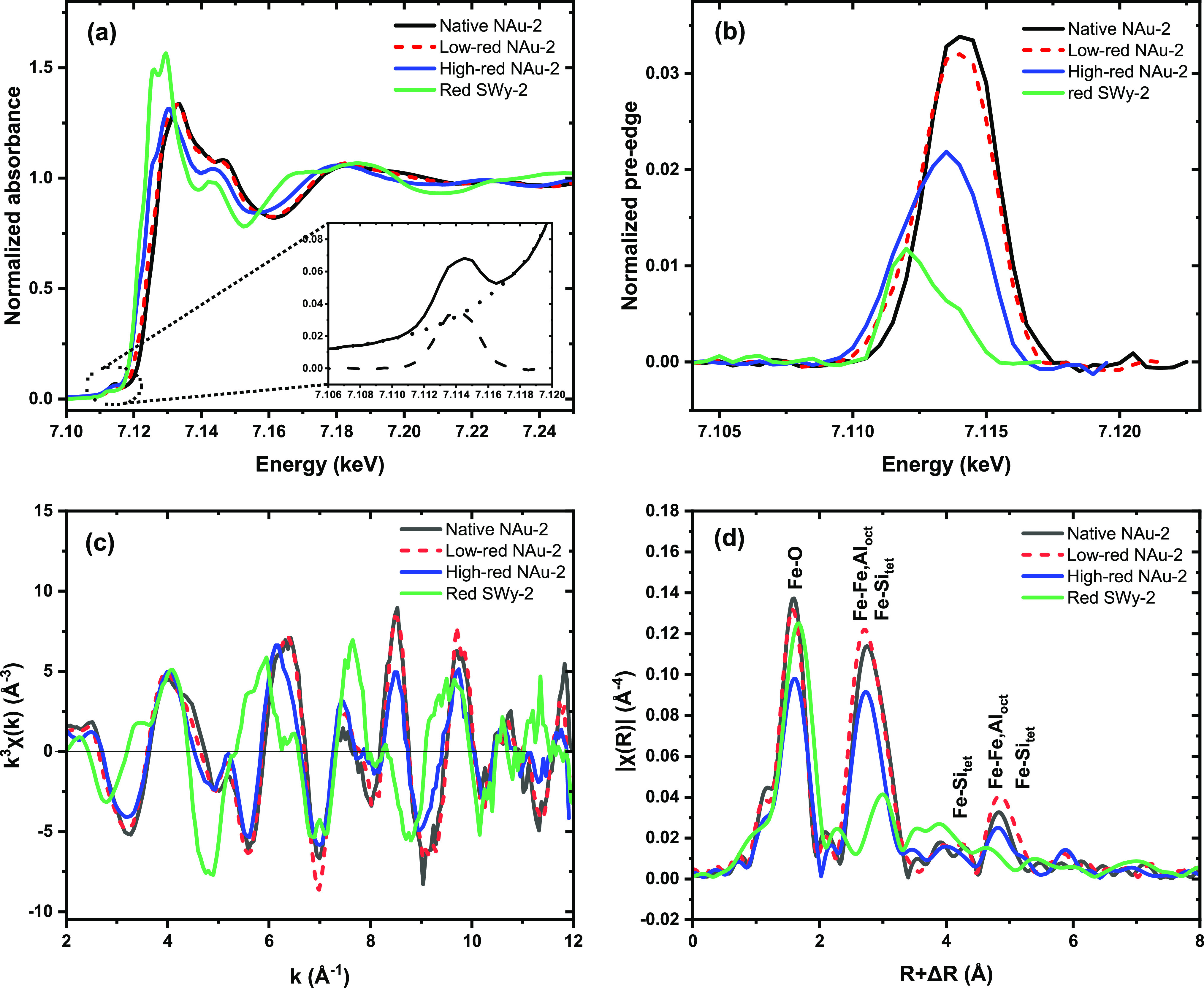
Fe K-edge XAFS spectra of native NAu-2, low-red NAu-2, high-red NAu-2, and red SWy-2. The spectrum of red SWy-2 is from Soltermann et al.,^74^ where the complete reduction of Fe was confirmed by Mössbauer spectrometry. (a) Normalized absorbance (XANES); the inset shows the pre-edge peak (solid line) of native NAu-2 with background (dotted line) and the separated pre-edge peak (dashed line). (b) normalized Fe K pre-edge peaks. (c) k^3^-weighted EXAFS chi function. (d) k^3^-weighted EXAFS Fourier transform magnitude.

Figure 6a shows the Fe K-edge XANES spectra. Assuming that Fe resides prevailingly in octahedral sites, the main absorption edge is shifted toward higher energy with increasing oxidation state because of the increasing shielding of the core electron. Accordingly, the edge of the clay samples increases as expected from the (fully reduced) SWy-2 to high-red NAu-2, low-red NAu-2, to the (fully oxidized) native NAu-2. To quantify the Fe(II) fraction, we first applied classical linear combination fit of the XANES spectra. As the first endmember we employed native NAu-2, where Mössbauer spectrometry and the Fe–O distance from EXAFS shell fitting (see below) confirmed 100% structural Fe(III). As the second endmember we tested first the XANES spectrum of an Fe(II) aquo complex, which provided only a poor reconstruction of the spectrum of high-red NAu-2, in spite of an assumed octahedral coordination to water molecules. A much better reconstruction was obtained by using the spectrum of red-SWy-2 from Soltermann et al. as the endmember,^74^ where the much lower content of structural Fe (2.9 wt%) permitted a complete Fe reduction as confirmed by Mössbauer spectrometry (Table S2 and Figure S2). The such obtained Fe(II) fractions of 0.14 for low-red NAu-2 and 0.48 for high-redNAu-2 are very similar to the Mössbauer values of 0.19 and 0.44 considering the intrinsic error of both methods (Table 1). For comparison, we analyzed the set of spectra also with the iterative transformation factor analysis (ITFA) package, which offers a more thorough statistical approach with respect to the number of spectral components present, as well as an improved noise filtering in comparison to linear combination fitting.^63^ Principal component analysis and the Malinoswki indicator confirmed that indeed only two components are present in the data set. Second, VARIMAX rotation and iterative target test transformation were applied to derive the fraction of Fe(II) in the samples. In spite of the presumably more robust procedure and the improved noise filtering of this method,^75^ the results were identical to conventional LCF (Table S2).

Since the XANES edge is not only influenced by the oxidation state, but due to multiple scattering phenomena also by the local coordination geometry, we analyzed also the pre-edge which gives directly the energy of the 1s to 3d electronic transitions and is hence a more direct approach for determining the oxidation state of Fe.^76^ For this, we extracted the pre-edge peak from the normalized XANES by subtracting a spline background (Figure 6a demonstrates the procedure and Figure 6b shows the extracted pre-edge peaks). As expected, the peaks shift to higher energy with decreasing Fe(II) fraction. A more refined analysis was conducted following the approach of Wilke et al. by fitting two Voigt functions to the pre-edge peaks (Figure S3) and calculating the centroid position (Table S3).^62^ When plotting the centroid position versus the Mössbauer-derived Fe(III) fraction, we obtained a linear relationship (Figure S4) following the regression equation *y* = 586.7*x* – 4173. Note that the normalized pre-edge height of tetrahedral Fe is >0.2,^62^ hence 1 order of magnitude higher than that of the NAu-2 samples, confirming that both Fe(II) and Fe(III) reside prevalently (>90%) in octahedral sites. We conclude that both the main XANES edge as well as its pre-edge provide reliable measurements for the Fe(II)/Fe(III) ratio in the nontronite samples, since the local Fe coordination remains octahedral.

Using Fe K-edge EXAFS spectroscopy, which provides the short-range order around the X-ray absorbing Fe atoms, we studied the structural changes induced in nontronite by the different redox steps. The k^3^-weighted EXAFS spectra (Figure 6c) as well as their Fourier transform magnitudes (FTM) (Figure 6d), which can be considered as statistical (pseudo-) radial distribution functions around the X-ray absorbing Fe atoms, show changes of the oxygen coordination sphere (1st FTM peak at about 1.7 Å), the nearest cation neighbors such as octahedral Fe or Al and tetrahedral Si (2nd FTM peaks at about 2.8 Å), and next nearest cation neighbors octahedral Fe or Al and tetrahedral Si (3rd FTM peaks at about 4.9 Å, all uncorrected for phase shift). Since Fe(II) and Fe(III) have different ionic radii (0.78 versus 0.65 Å for six-coordinated cations in high-spin configuration^77^), the length of the Fe–O bond commonly decreases from e.g. 2.14 Å in Fe(OH)_2_ to about 2.00 Å in common Fe(III) (hydr)oxides. Note that the EXAFS resolution of 0.16 Å as given by the chi-range of 2 to 12 Å^–1^ did not allow us to fit the shells corresponding to octahedral Fe(II), Fe(III), and potentially mixed Fe(II,III) sites individually. Table 2 shows hence only the average distance determined by fitting one Fe-O shell. The fully oxidized standard for structural Fe(III), native NAu-2, has an Fe–O distance of 2.01 Å, close to that of Fe(III) (hydr-)oxides. The fully reduced standard for structural Fe(II), red SWy-2, has an Fe-O distance of 2.10 Å, hence 0.02 to 0.04 Å shorter than common Fe(II)-O coordinated structures, most likely due to constraints of the clay structure. Low-red NAu-2 has a distance of 2.02 Å and high-red NAu-2 2.05 Å (Table 2). Assuming a linear relationship between Fe oxidation state and Fe-O bond length within the octahedral clay sites, we obtain based on the two endmembers *y* = 10.99*x* – 22.09 (*y*: fraction of Fe(II), *x*: Fe-O bond length), and hence 11% Fe(II) for low reduced NAu-2 and 44% for high reduced NAu-2, in excellent agreement with the XANES result. Therefore, also EXAFS fitting of the Fe-O coordination shell can be used to derive the Fe oxidation state in nontronite and similar dioctahedral clays.

**Table 2:** Fe K-Edge EXAFS Shell Fitting Results^h^

sample	shell	CN^a^	*R* [Å]^b^	σ^2^ [Å^2^]^c^	Δ*E*_0_ [eV]	*S*_0_^2^	residual
Native NAu-2	Fe-O	6^d^	2.01	0.0042	10.1	0.57	9.7
	Fe-Fe	2.1^e^	3.04^e^	0.0010^e^			
	Fe-Al^g^	0.9^e^	3.04^e^	0.0010^e^			
	Fe-Si	4^d^	3.28	0.0010			
	Fe-Si	4^d^	4.46	0.0046			
	Fe-Fe	4.5^f^	5.26^f^	0.0042^f^			
	Fe-Al^g^	1.5^f^	5.26^f^	0.0042^f^			
	Fe-Si	8^d^	5.54	0.0066			
Low-red NAu-2	Fe-O	6^d^	2.02	0.0039	5.8	0.56	6.2
	Fe-Fe	2.5^e^	3.04^e^	0.0012^e^			
	Fe-Al^g^	0.5^e^	3.04^e^	0.0012^e^			
	Fe-Si	4^d^	3.28	0.0010			
	Fe-Si	4^d^	4.48	0.0028			
	Fe-Fe	5.5^f^	5.25^f^	0.0042^f^			
	Fe-Al^g^	0.5^f^	5.25^f^	0.0042^f^			
	Fe-Si	8^d^	5.52	0.0044			
High-red NAu-2	Fe-O	6^d^	2.05	0.0066	6.4	0.56	7.8
	Fe-Fe	2.5^e^	3.05^e^	0.0039^e^			
	Fe-Al^g^	0.5^e^	3.05^e^	0.0039^e^			
	Fe-Si	4^d^	3.30	0.0029			
	Fe-Si	4^d^	4.46	0.0039			
	Fe-Fe	5.5^f^	5.23^f^	0.0081^f^			
	Fe-Al^g^	0.5^f^	5.23^f^	0.0081^f^			
	Fe-Si	8^d^	5.49	0.0091			
Red SWy-2	Fe-O	6^d^	2.10	0.0031	6.64	0.70	4.6

a Error of coordination number (CN) ± 25%.

b Error of radial distance (*R*) ± 0.01 Å.

c Error of Debye–Waller factor (σ^2^) ± 0.002 Å.

d CN fixed according to the structural model of Garfield nontronite.^39^

e For the fits of the two nearest Fe_Oct_ and Al_Oct_ paths, the sum of both CNs was fixed at 3, while both their *R* and σ^2^ values were correlated.

f For the fits of the next nearest Fe_Oct_ and Al_Oct_ paths, the sum of both CNs was fixed at 6, while both their *R* and σ^2^ values were correlated.

g Fe-Al represents Fe-(Al+Mg). Al and Mg cannot be differentiated by EXAFS due to their similar electron densities.

h Fitted spectra are shown in Figure S5.

Even more importantly, EXAFS shell fitting allowed us to study the structural changes induced by the reduction within radial distances up to 6 Å from the Fe centers. In native NAu-2, 73% of octahedral positions are occupied by Fe, and the remaining 27% by Al and Mg, as indicated by the structural equation (M^+^_0.97_)[Si_7.57_Al_0.01_Fe_0.42_][Al_0.52_ Fe_3.32_ Mg_0.7_]O_20_(OH)_4_.^48^ To reflect this situation during our EXAFS shell fits, we fitted first and second metal shells using both Fe-Fe and Fe-Al paths (note that both Al and Mg produce almost identical backscattering functions and hence cannot be distinguished). To reduce the large number of fitting parameters, which would necessarily lead to strong collinearity effects, we correlated distances and Debye–Waller factors for both elements and limited the sum of the coordination numbers to their crystallographic values of the dioctahedral structure, i.e. 3 for the first and 6 for the second metal shell (Table 2). The such derived first metal shell coordination numbers of 2.1 for Fe and 0.9 for Al/Mg correspond to octahedral occupancies of 70% Fe (2.1/3.0*100) and 30% Al/Mg (0.9/3.0*100), thereby nicely confirming the independently derived structural equation of nontronite in spite of the rather high error for EXAFS-derived coordination numbers.

For the two reduced samples, the coordination numbers of the two Fe-Fe shells increase, while those of the Fe-Al shells decrease. For the first metal shell, we thereby obtain a Fe occupancy of 83% (2.5/3*100), and correspondingly of 17% for Al/Mg (Table 2). These changes suggest the formation of Fe clusters by the clay reduction, in line with previous results by Manceau et al., who proposed a migration of Fe cations to neighboring sites after the reduction of Fe(III) to Fe(II).^45^ These authors also suggested that this clustering of Fe(II) sites is accompanied by formation of a local trioctahedral structure. Due to model overparametrization problems (see above), we were not able to adjust our EXAFS fitting scheme to allow for such a transition, which would require a fitted increase of the summed coordination number of the first Fe-Fe,Al shell to a range from 3 to 6. Note that the fitted S_0_^2^ values of the clay samples are with 0.56 rather small due to fluorescence self-absorption effects because of the rather high Fe concentrations, but they did not influence the coordination numbers, which were fixed according to the crystallographic nontronite model.

The obtained distances of native NAu-2 are well in line with the nontronite structure. For high-red NAu-2, the nearest Fe-Fe,Al and Fe-Si shells show a slight increase of 0.01 to 0.02 Å, probably an effect of the average increase of Fe-O coordination distances due to an increase of the Fe(II) fraction. Note, however, that this tendency is not confirmed for longer distances. The Debye–Waller factors of all shells reveal an increasing static disorder of the shells with increasing Fe(II)/Fe(III) ratio. This is coherent with an increasing degree of structural constraints in the layers due to isomorphous substitution of Fe(III) by Fe(II).

### 3.7 Mediated Electrochemical Oxidation and Reduction

In the clay sample, Fe(II) can be transformed to Fe(III) and vice versa by applying oxidizing and reducing potential, respectively. Mediators are used to facilitate the electron transfer in the redox reaction between structural Fe in the clay minerals and working electrode. When an potential is applied, the amount of electrons transferred can be calculated by integration of the generated current peak so as to get the amount of Fe(II) or Fe(III) in the clay mineral. Table 3 shows that the Fe(II)% of native, low-red, and high-red NAu-2 are 0.1%, 19%, and 58%. Moreover, the sum of EDC and EAC indicates the amount of redox-active Fe in the clay structure. In both native and reduced NAu-2 samples, around 30% of total structural Fe is not redox-active when measured by MEO/MER. The fact that 20% of total structural Fe in nontronite is not redox-active has also been reported before by Gorski et al.^65^

**Table 3:** EDC and EAC of Nontronite Clay Samples Measured by MEO/MER

Sample	total Fe^b^ (mmol/g_clay_)	EDC^a^ (mmol_e-_/g_clay_)	EAC^a^ (mmol_e-_/g_clay_)	EDC (% Fe_tot_)	EAC (% Fe_tot_)
Native NAu-2	3.9	0.00	2.94	0.1	75
Low-red NAu-2	3.8	0.74	1.72	19	45
High-red NAu-2	3.8	2.19	0.45	58	12

a Electron donating capacity (EDC, mmol_e-_/g_clay_) and electron accepting capacity (EAC, mmol_e-_/g_clay_) are the amount of Fe(II) and Fe(III) normalized with the mass of clay mineral, respectively.

b The total Fe amount was corresponding to 22 wt% Fe in the clay structure.

### 3.8 Fe Redox State Identification

Different methods were applied to measure the Fe redox state in nontronite. XAS demonstrates relatively lower Fe(II) fractions on low-red NAu-2 compared with the results of MEO/MER and Mössbauer spectrometry; the Fe(II) fractions of high-red NAu-2 is between the results of Mössbauer spectrometry and MEO/MER. Meanwhile, XPS shows slightly higher Fe(II) fractions on both reduced samples comparing with Mössbauer result (Table 4). First of all, the sample preparation of each method has been conducted with uttermost care to keep the original oxidation state. Schaefer et al. suggested that Mössbauer spectra measured at RT and even at 77 K may significantly underestimate Fe(II);^85^ we cannot confirm this, since our data collected at 77 K agree well with that of the other techniques. X-ray irradiation induced Fe reduction in XPS was evident since 0.07 of Fe(II) fraction was detected in native NAu-2, which in fact should be fully oxidized. Sorption of cation mediators on montmorillonite has been reported owing to the high surface charge density of clay minerals and the increase of the current peak in MEO/MER measurement was substantial;^78^ nevertheless, ABTS is an anion mediator, of which sorption is not an issue in the measurement. Little has been known so far concerning the stability of mediators and the high Fe(II) fraction 0.58 in high-red NAu-2 in MEO/MER measurement. Both XANES and EXAFS as independent measurements of prevalently electronic and short-range structure, respectively, show good consistency of Fe(II) fractions. The results confirm the reliability of the different analyses of XANES and EXAFS; however, they are also highly dependent on the calibration curve given by standards.^62,79^ Initially, Fe^2+^_aq_ was utilized as a reference for Fe(II) standard; however, it exhibited an approximately 8% difference in Fe(II) fraction owing to dissimilar structures compared to smectite clays. Hence, careful consideration is necessary when selecting standards to establish a precise calibration curve before determining Fe(II) fractions. Overall, the Mössbauer measurement maintains its rank as the currently most reliable stand-alone method to determine the Fe(II) fraction in clay minerals.

**Table 4:** Comparison of Fe(II) Fractions in the Three NAu-2 Samples Obtained by Different Measurements

	Mössbauer at 77 K	XPS	XAS				MEO/MER
			XANES centroid	XANES ITFA	XANES LCF	EXAFS Fe-O	
Native NAu-2	0.00	0.07	0.00^a^	0.00^a^	0.00^a^	0.00^a^	0.00
Low-red NAu-2	0.19	0.24	0.16	0.15	0.14	0.11	0.19
High-red NAu-2	0.44	0.46	0.50	0.51	0.48	0.44	0.58

a Note that for the XANES centroid and EXAFS Fe-O methods, native NAu-2 was used as the standard for pure Fe(III), and hence Fe(II) fractions of this sample are necessarily 0, while Mössbauer, XPS, and MEO/MER provide the Fe(II) fraction of native NAu-2 independently.

## 4 Conclusions

In this study, we demonstrate the structure of our native and reduced nontronite samples, and the structural integrity is ensured after CBD reduction. Quantitative Fe redox state measurements including Mössbauer spectrometry, XPS, XANES, EXAFS, and MEO/MER are compared to exhibit the difference between each method. All methods provide errors of only a few percent relative to a mean value, and this is most likely because of the large precautions to conserve the Fe oxidation state from clay reduction to final analysis. The consistent results suggest that all methods provide reliable Fe(II)/Fe(III) ratios and can be applied to other clay samples.

Fe-bearing clay minerals are one of the main components in soil. Variation of redox potential induced by Fe-bearing clay minerals under alternate flooding-drining condition is expected in different soils and can cause significant changes in soil functioning, plant response, soil bacterial community, N and P removal efficiency, organic matter decomposition, nutrient cycling, etc.^80−83^ Therefore, comprehensive characterization methods of structural Fe redox state associated with redox potential are of particular importance in monitoring and predicting the biogeochemical processes in the natural environment. Moreover, Fe-bearing clay minerals also have a strong influence on the fate of contaminants in the environment. Especially in deep geological repositories, Fe-bearing clay minerals play an important role in controlling and immobilizing the radionuclides in radioactive waste by reducing them to a lower and less soluble valence state. A thorough comprehension of these redox processes can significantly contribute to the safety assessment.^78,84^ Such a complete picture of the nontronite structure, reduction, and Fe redox state identification builds up a strong basis for studies on Fe-bearing clay minerals and serves as a bench mark test for Fe redox state measurements in other clays.

## Supporting Information Available

Photographs of native NAu-2, high-red NAu-2, and low-red NAu-2 clay suspensions; XPS fitting results; ITFA and LCF fitting results; LCF fitting spectra; figures of Voigt deconvolution of normalized pre-edge; results of Voigt deconvolution of normalized Fe-K Pre-edge peaks; correlation between Mössbauer-determined Fe(III) fraction and pre-edge centroid position and Fe–O bond length; and Fe K-edge EXAS fitted spectra (PDF)
