# Supplementary material for: Oxidation State and Structure of Fe in Nontronite: From Oxidizing to Reducing Conditions
Source: ACS Earth Space Chem. 2023 Sep 27;7(10):1868–81. doi: 10.1021/acsearthspacechem.3c00136 (PMC10594735; doi:10.1021/acsearthspacechem.3c00136)
Supplement: Supplementary file 1 — sp3c00136_si_001.pdf [file sp3c00136_si_001.pdf]

Supporting Information

Oxidation state and structure of Fe in nontronite: from oxidizing to reducing conditions

Yanting Qian[1,2] , Andreas C. Scheinost[3a,b], Sylvain Grangeon[4], Jean-Marc Greneche[5], Alwina Hoving[6], Eric Bourhis[7], Nicolas Maubec[4], Sergey V. Churakov[1,2], Maria Marques Fernandes[1]

[1]Laboratory for Waste Management, Paul Scherrer Institut, CH-5232 Villigen PSI, Switzerland

[2]Institute for Geological Sciences, University of Bern, CH-3012 Bern, Switzerland

[3a]The Rossendorf Beamline at the European Synchrotron Radiation Facility (ESRF), Avenue des Martyrs 71, 38043, Grenoble, France

[3b]Helmholtz Zentrum Dresden Rossendorf, Institute of Resource Ecology, Bautzner Landstrasse 400, 01328, Dresden, Germany

[4]BRGM – French Geological Survey, 45060 Orléans, France

[5]Institut des Molécules et Matériaux du Mans IMMM UMR CNRS 6283, Le Mans Université, 72085, Le Mans Cedex 9, France

[6]TNO Geological Survey of the Netherlands, PO Box 80015, 3508 TA Utrecht, the Netherlands

[7]Interfaces, Confinement, Matériaux et Nanostructures (ICMN), CNRS/Université d'Orléans, UMR 7374, 1b rue de la Férollerie, CS 40059, 45071 Orléans, France

This supporting information contains 5 figures, and 3 tables.

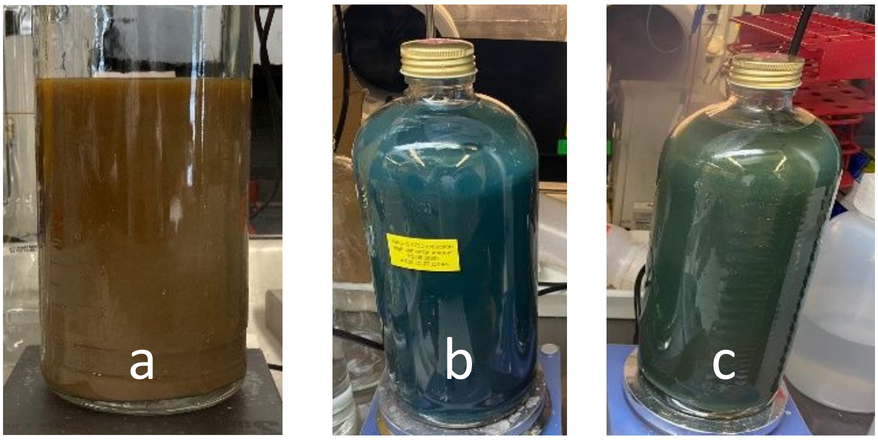

Figure S1. Photographs of nontronite clay suspensions stored in infusion bottles in the glovebox. (a) Native NAu-2, (b) high-red NAu-2, (c) low-red NAu-2.

Table S1. XPS fitting results of native NAu-2, low-red NAu-2, and high-red NAu-2.

Sample	$Fe2p_{3/2}$ species	Binding energy (eV)	FWHM (eV)	Area (cps·eV)	Atomic %
Native NAu-2	Fe(II)-O1	708.7	1.50	388	1.31
	Fe(II)-O2	709.5	1.70	545	1.85
	Fe(II)-O3	711.1	1.70	264	0.89
	Fe(II)-O4	711.9	2.82	724	2.46
	Fe(II)-O5	715.2	2.30	137	0.46
	Fe(III)-O1	711.6	1.75	12629	42.84
	Fe(III)-O2	712.8	1.95	8448	28.67
	Fe(III)-O3	713.8	1.95	4224	14.34
	Fe(III)-O4	715.0	1.95	2112	7.17
Low-red NAu-2	Fe(II)-O1	708.9	1.50	2601	4.58
	Fe(II)-O2	709.5	1.70	3653	6.43
	Fe(II)-O3	711.1	1.70	1768	3.11
	Fe(II)-O4	711.9	2.82	4854	8.55
	Fe(II)-O5	715.2	2.30	917	1.62
	Fe(III)-O1	712.0	1.75	19791	34.87
	Fe(III)-O2	713.1	1.95	13238	23.33
	Fe(III)-O3	714.2	1.95	6619	11.67
	Fe(III)-O4	715.1	1.95	3310	5.84
High-red NAu-2	Fe(II)-O1	708.7	1.50	4908	8.67
	Fe(II)-O2	709.9	1.70	6895	12.19
	Fe(II)-O3	711.1	1.70	3336	5.90
	Fe(II)-O4	711.9	2.82	9161	16.20
	Fe(II)-O5	715.2	2.30	1730	3.06
	Fe(III)-O1	711.6	1.75	14054	24.86
	Fe(III)-O2	712.8	1.95	9401	16.64

	Fe(III)-O3	713.8	1.95	4700	8.32
	Fe(III)-O4	714.8	1.95	2350	4.16

Table S2. Fitting results of the Fe-K main-edge in Figure 6a by ITFA (non-normalized to unity) and by linear combination fitting (LCF, normalized to unity) using native NAu-2 as standard for Fe(III) and red SWy-2 as standard for Fe(II).

Sample	ITFA		LCF		
	Fe(III)	Fe(II)	Fe(III)	Fe(II)	R factor
Native NAu-2	1.00	0.00	1.00	0.00	
Low-red NAu-2	0.86	0.15	0.86	0.14	0.0005
High-red NAu-2	0.47	0.51	0.52	0.48	0.0051

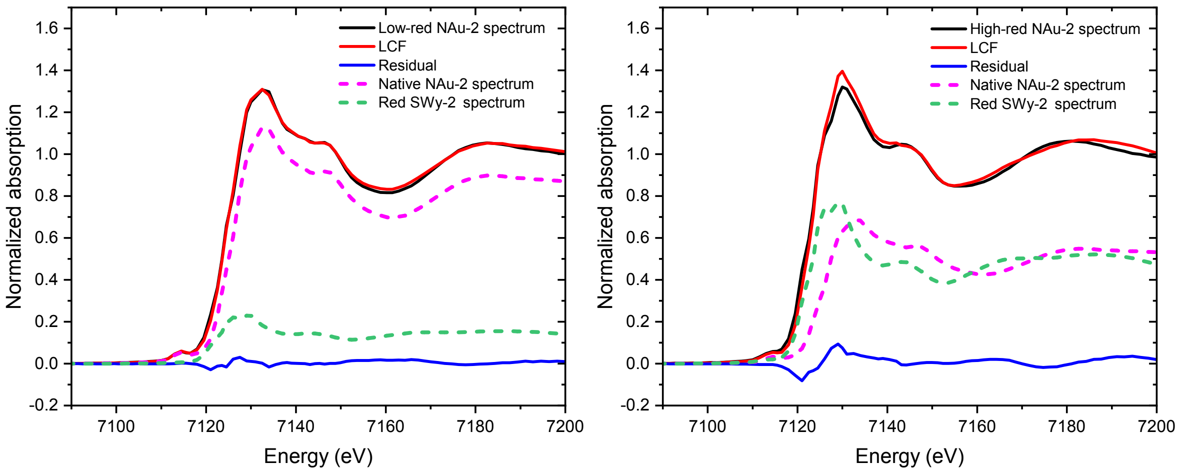

Figure S2. Linear combinination fit of (a) low-red NAu-2 and (b) high-red NAu-2 spectra using the XANES spectra of native NAu-2 and red SWy-2 as standards for structural Fe(III) and Fe(II), respectively.

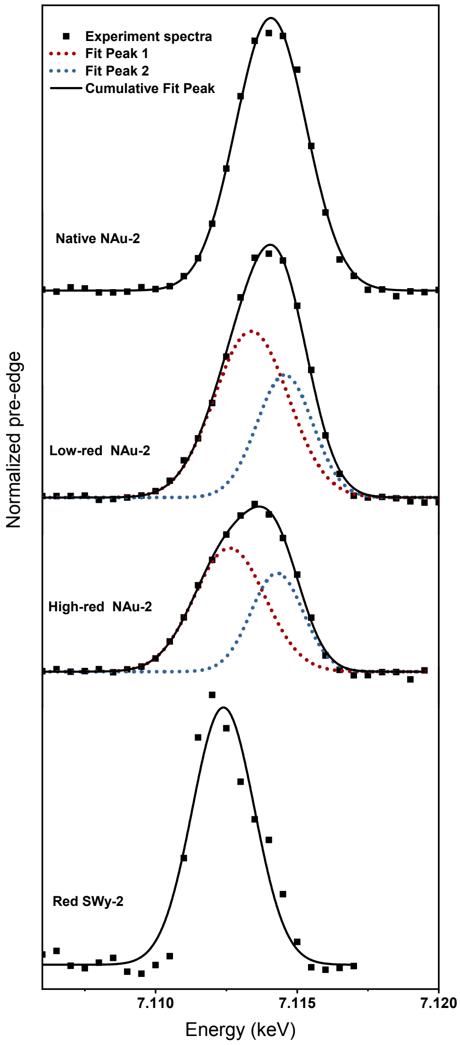

Figure S3. Voigt deconvolution of normalized pre-edg.

Table S3. Voigt deconvolution of normalized Fe-K pre-edge peaks.

Sample	Peak position (keV)	FWHM (keV)	Area	Total area	Centroid (keV)	R^2
Native NAu-2	7.11407	0.00293	1.12E-04	1.12E-04	7.11407	0.9964
Low-red NAu-2	7.11338	0.00308	7.19E-05	1.12E-04	7.11380	0.9975
	7.11456	0.00231	3.99E-05			
High-red NAu-2	7.11261	0.00295	5.12E-05	8.13E-05	7.11323	0.9971
	7.11429	0.00217	3.01E-05			
Red SWy-2	7.11240	0.00258	4.58E-05	3.08E-05	7.11240	0.9391

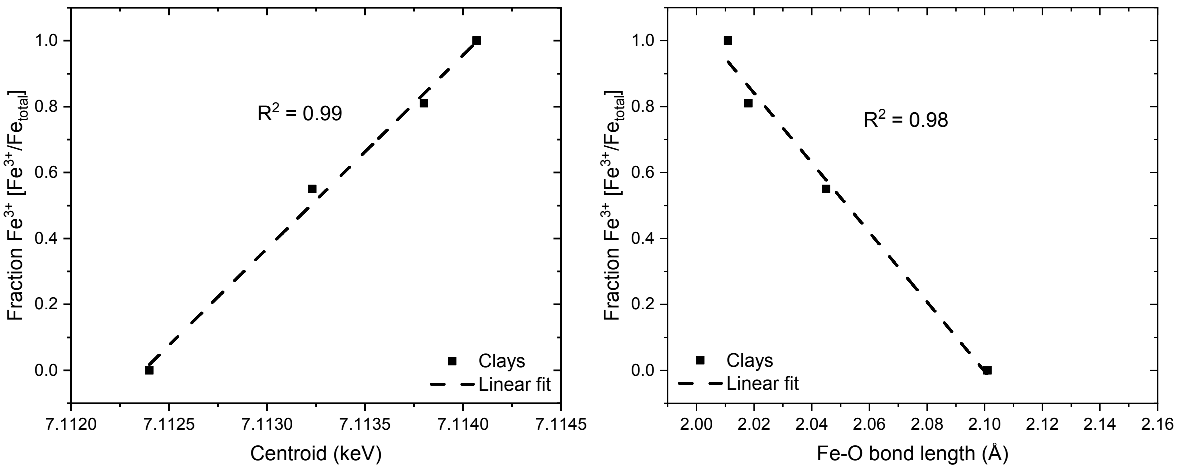

Figure S4. (a) Correlation between Mössbauer-determined Fe(III) fraction (y-axis) and pre-edge centroid (x-axis) with regression equation y=586.7x-4173. (b) Correlation between Mössbauer measured Fe(III) fraction (y-axis) and Fe-O bond length derived by EXAFS shell fit (x-axis) bond length with regression equation y=-10.6x+22.2.

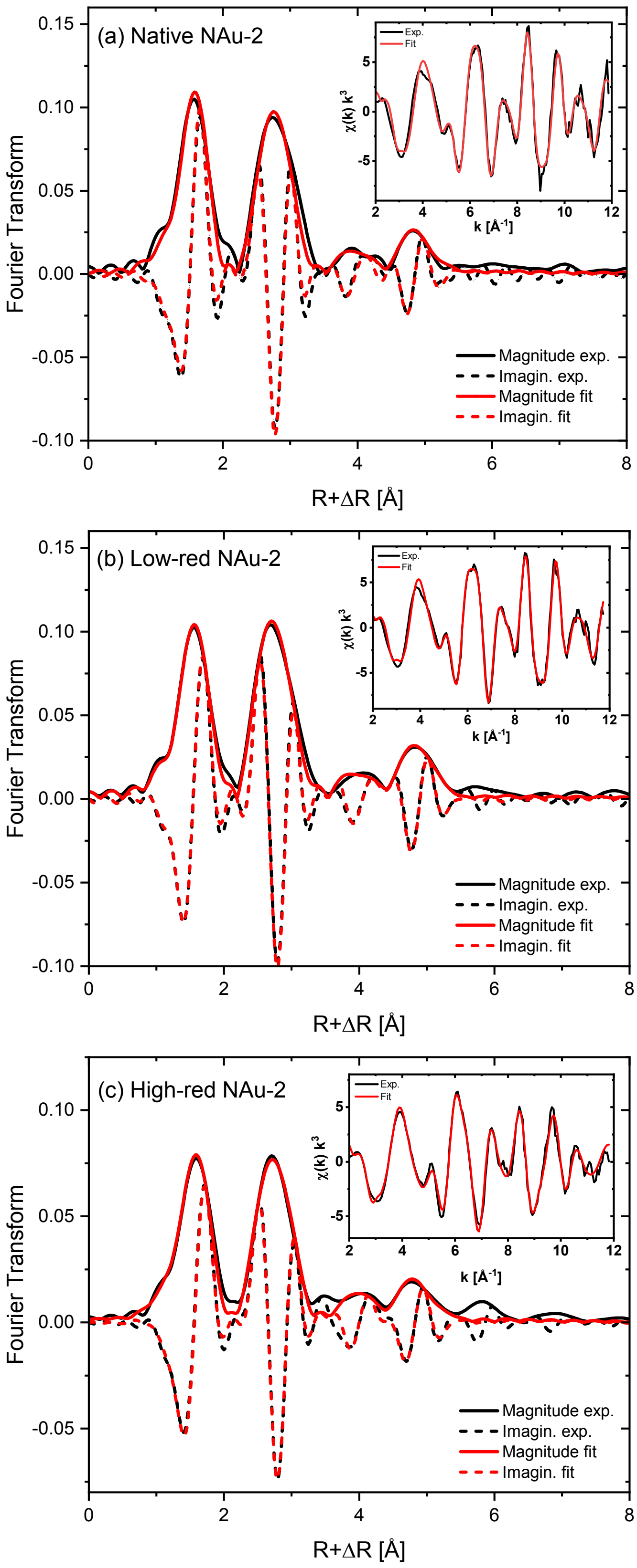

Figure S5. Fe K-edge EXAFS fitted spectra.
